# Metabolic diseases and healthy aging: identifying environmental and behavioral risk factors and promoting public health

**DOI:** 10.3389/fpubh.2023.1253506

**Published:** 2023-10-13

**Authors:** Kexin Zhang, Yujie Ma, Youhong Luo, Yixin Song, Guoji Xiong, Yanhui Ma, Xiaodong Sun, Chengxia Kan

**Affiliations:** ^1^Department of Endocrinology and Metabolism, Affiliated Hospital of Weifang Medical University, School of Clinical Medicine, Weifang Medical University, Weifang, China; ^2^Clinical Research Center, Affiliated Hospital of Weifang Medical University, Weifang, China; ^3^Department of Pathophysiology, School of Basic Medical Sciences, Weifang Medical University, Weifang, China; ^4^Department of Pathology, Affiliated Hospital of Weifang Medical University, Weifang, China

**Keywords:** metabolic diseases, healthy aging, risk factors, public health, environment

## Abstract

Aging is a progressive and irreversible pathophysiological process that manifests as the decline in tissue and cellular functions, along with a significant increase in the risk of various aging-related diseases, including metabolic diseases. While advances in modern medicine have significantly promoted human health and extended human lifespan, metabolic diseases such as obesity and type 2 diabetes among the older adults pose a major challenge to global public health as societies age. Therefore, understanding the complex interaction between risk factors and metabolic diseases is crucial for promoting well-being and healthy aging. This review article explores the environmental and behavioral risk factors associated with metabolic diseases and their impact on healthy aging. The environment, including an obesogenic environment and exposure to environmental toxins, is strongly correlated with the rising prevalence of obesity and its comorbidities. Behavioral factors, such as diet, physical activity, smoking, alcohol consumption, and sleep patterns, significantly influence the risk of metabolic diseases throughout aging. Public health interventions targeting modifiable risk factors can effectively promote healthier lifestyles and prevent metabolic diseases. Collaboration between government agencies, healthcare providers and community organizations is essential for implementing these interventions and creating supportive environments that foster healthy aging.

## 1. Introduction

Aging is an ongoing and irreversible physiological process that leads to the gradual deterioration of tissue and cellular functions, increasing the susceptibility to age-related diseases, including metabolic disorders (1). While remarkable advancements in modern medicine have improved human health and extended lifespan, metabolic conditions like obesity and type 2 diabetes (T2D) continue to pose significant global public health challenges as populations age (1–5). Understanding the complex relationship between metabolic diseases and healthy aging is crucial for promoting well-being and preventing disease burden. While genetics play a role in metabolic disease development, environmental and behavioral factors also contribute significantly (6–10). An obesogenic environment, characterized by air pollution, pesticides and exposure to environmental toxins, correlates strongly with the rising prevalence of obesity and its associated comorbidities (8, 10–14). Additionally, research suggests that behavioral factors, such as dietary choices, physical activity levels, and sleep patterns, significantly influence the risk of metabolic diseases throughout aging (15–18).

**Figure 1:**
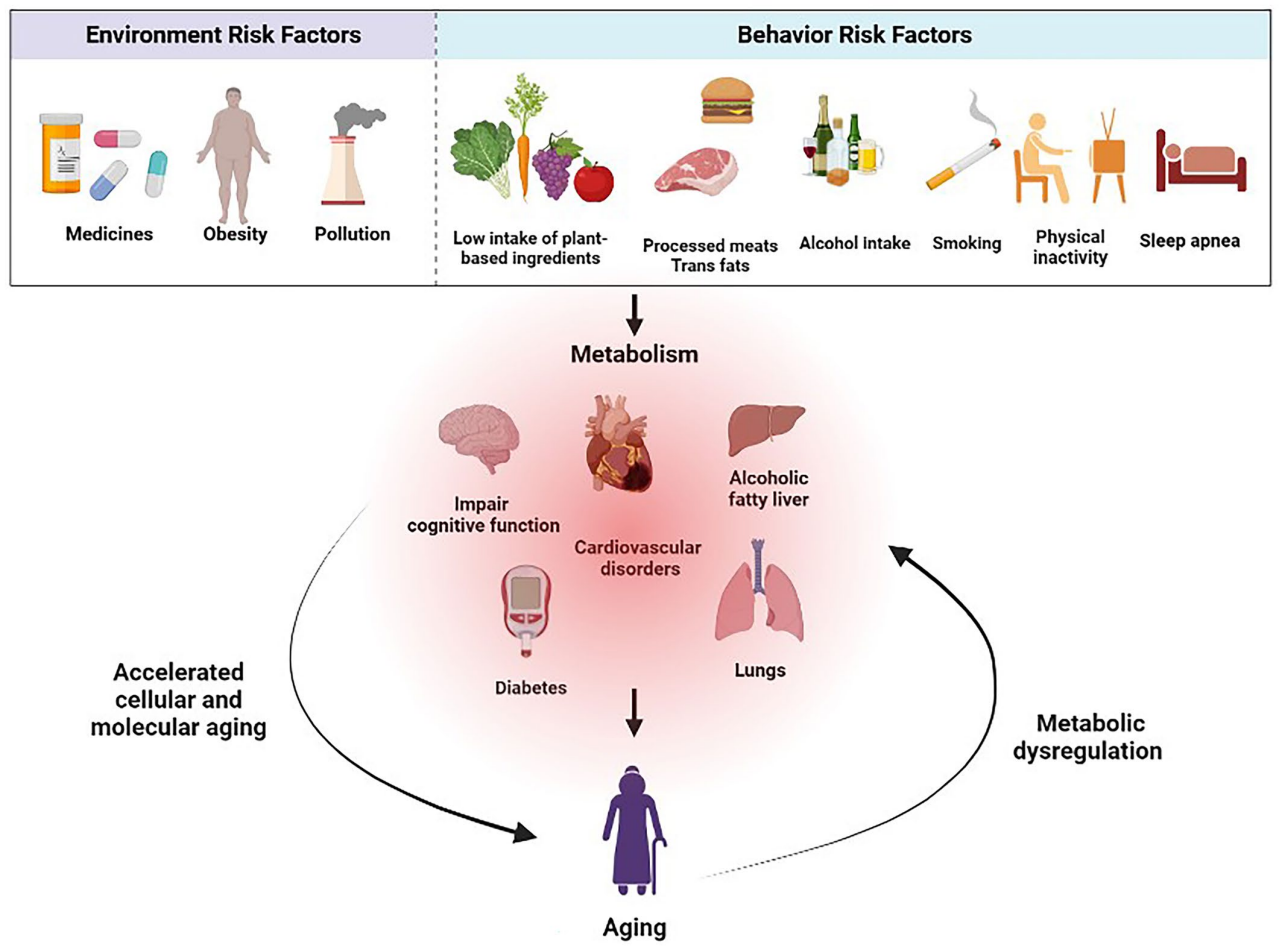
The interactions between aging and metabolic diseases.

Effective strategies must be implemented to promote healthy aging and prevent metabolic diseases. This involves understanding the underlying risk factors and their impact on public health, which can then inform the development of targeted interventions and policies that encourage healthier lifestyles. Public health initiatives can address these risks to promote healthier choices among the population by identifying modifiable environmental and behavioral factors (Figure 1).

This review article aims to explore the intricate relationship between metabolic diseases and healthy aging by examining the environmental and behavioral risk factors contributing to their onset and progression. By analyzing current research and existing literature, we will delve into the multifaceted nature of these diseases, considering factors such as diet, physical activity, stress, sleep patterns, and socioeconomic determinants. Furthermore, we will explore the potential of public health interventions to mitigate the impact of metabolic diseases and promote healthy aging (Table 1).

**Table 1:** Risk Factors and their relationship with metabolic diseases and aging.

Risk factors	Relationship to metabolic diseases	Relationship to aging	Refs
Environmental pollutants	Increase metabolic diseases risk	Accelerate the aging process	(12, 13, 50–52)
Diet and nutrition	Poor dietary choices contribute to metabolic diseases; a healthy diet can maintain optimal metabolic function	Poor diet can aggravate metabolic diseases and aging; healthy diet can maintaining promoting healthy aging	(69–74, 161)
Physical activity and sedentary behavior	Regular physical activity can reduce metabolic diseases risk; Sedentary lifestyle can elevate metabolic diseases risk	Regular physical activity can delay aging; Sedentary can promote aging	(35, 92, 93, 99, 100)
Tobacco smoking	Increase metabolic diseases risk	Accelerate the aging process	(120, 121, 123–128)
Alcohol consumption	Excessive and prolonged alcohol consumption can have detrimental effects on metabolic health	Excessive and long-term alcohol consumption has a negative impact on aging	(131–136)
Sleep patterns and quality	Chronic sleep deprivation, poor sleep quality, and disturbances in sleep patterns elevate metabolic diseases risk	Inadequate sleep can impair cognitive function and accelerates the aging-related complications	(140–146)
Physical stress and mental health	Increase metabolic diseases risk	Accelerate the aging process	(147–153, 162)

## 2. The interactions between aging and metabolic diseases

### 2.1. Aging as a risk factor for metabolic diseases

Aging is a significant risk factor for developing and progressing metabolic diseases in older adults due to various physiological changes that occur with age (19, 20). These changes affect metabolic regulation and contribute to the increased risk of metabolic disorders. One notable change associated with aging is the decline in metabolic rate. If calorie intake remains constant or increases, this decline in metabolic rate can lead to weight gain, obesity and insulin resistance (IR) (21). IR refers to repaired responses to insulin stimulation in specific tissues, primarily the liver, muscles, and adipose tissues. This impairment leads to ineffective glucose utilization, prompting a compensatory increase in β-cell insulin production and ultimately resulting in hyperinsulinemia (22, 23). This age-related decline in insulin sensitivity becomes more prominent as people grow older. Moreover, aging affects body composition, leading to increased adiposity (fat accumulation) and decreased lean muscle mass (24). This shift in body composition, known as sarcopenic obesity, contributes to metabolic dysregulation and raises the risk of metabolic diseases (24, 25). Besides these factors, aging-related hormonal changes play a role in metabolic dysregulation. Aging is accompanied by a natural decline in growth hormone and insulin-like growth factor-1 (IGF-1) levels (26, 27). This reduction increases adiposity, particularly abdominal fat, while decreasing muscle mass. Lower hormone levels lead to IR, glucose intolerance (the body’s struggle to control blood glucose levels, resulting in elevated levels not yet classified as diabetes), and the potential development of diabetes (28, 29). In women, menopause triggers a substantial decrease in estrogen levels, potentially resulting in IR and an increase in abdominal fat, since estrogen renders tissues more sensitive to insulin (30, 31). Similarly, men experience a gradual decline in testosterone levels with aging, resulting in higher body fat levels, loss of muscle mass, and IR (32). These hormonal changes influence fat metabolism, insulin sensitivity, and energy expenditure, thereby increasing the risk of metabolic diseases in older adults (33, 34).

Furthermore, aging is associated with a higher prevalence of additional risk factors for metabolic diseases. Reduced physical activity, sedentary behavior, underlying genetic predispositions, and cumulative exposure to environmental factors over a lifetime increase risk in older adults (17, 35). When combined with age-related changes in metabolic regulation, these factors contribute to the bidirectional relationship between aging and metabolic diseases (6, 8, 10).

### 2.2. Impact of metabolic diseases on the aging process

Metabolic diseases, particularly diabetes and hypertension, significantly impact aging by accelerating age-related decline in health and functionality (19, 20). Both conditions foster systemic inflammation and oxidative stress, contributing to cellular and molecular aging (36–39). Chronic inflammation from these diseases elevates reactive oxygen species levels, inflicting cellular damage and disrupting normal functions. It also induces IR, which inhibits glucose uptake by cells and raises blood glucose levels, thereby impairing glucose metabolism and leading to complications like renal disease and cognitive decline (40–43). Diabetes causes microvascular and macrovascular damage, increasing the risk of complications such as diabetic retinopathy, peripheral neuropathy, and cardiovascular issues. These conditions impede sensory and motor functions, limit mobility, and negatively affect overall well-being (44–47). Hypertension, often related to metabolic disorders like obesity and IR, imposes strain on the cardiovascular system, accelerating aging and promoting heart-related complications (38, 39). It also exacerbates organ damage, leading to chronic conditions like kidney disease, dementia, and heart failure, thereby further speeding up the aging process when coupled with metabolic syndrome (42, 43, 48, 49).

## 3. Environment risk factors

Environmental pollutants, including air pollutants, pesticides, heavy metals, and endocrine-disrupting compounds (EDCs), pose substantial risks to metabolic health and can accelerate aging (12, 50, 51). Air pollution, especially fine particulate matter (PM2.5) and nitrogen dioxide has been linked to IR, inflammation, and an increased risk of metabolic disorders such as obesity, T2D, and cardiovascular disease (CVD) (13, 52). Similarly, pesticides can disrupt metabolic processes and hormonal regulation, increasing the risk of diseases like IR and obesity (12, 14). Heavy metals, such as lead, cadmium, and mercury, can also induce metabolic dysfunction and oxidative stress (50).

EDCs are present in various consumer products and can interfere with the production, transport, or metabolism of hormones. It is estimated that there are more than one thousand different EDCs. EDCs disrupt hormonal action, while metabolic disruptors affect metabolism. It is crucial to clarify that not all metabolic disruptors are EDCs. EDCs specifically pertain to substances that interfere with the hormonal aspects of metabolism (51, 53–56). Therefore, mitigating exposure to these pollutants can help reduce the risk of metabolic diseases and age-related complications.

The term “exposome” encompasses both external and internal environmental factors that impact human health. It includes the ecto-exposome, which pertains to external environmental influences, and the endo-exposome, which relates to the immediate adjacent extracellular environment. Both the ecto-exposome and the endo-exposome are conditions that support cell survival and organ/system function. According to reports, persistent organic pollutants, also known as ‘EDCs,’ interfere with the endocrine system and are found in the environment, causing lifelong exposure in certain populations (57, 58). Occupational exposure to toxins, including heavy metals, solvents, pesticides, and industrial chemicals, is a significant risk factor for metabolic diseases and healthy aging (14, 59–61). The heavy use of these toxins in industries such as manufacturing, agriculture, and mining can lead to adverse health effects like IR, dyslipidemia, impaired glucose metabolism, hormonal disruption, and altered lipid metabolism (62–64). Toxic exposure can also influence aging by promoting inflammation, DNA damage, mitochondrial dysfunction, and impairing natural defense mechanisms against oxidation, thereby accelerating cellular aging and increasing the risk of age-related diseases (12, 65–68). However, little is known about how these exposures actually affect humans or potentially affect the onset of metabolic diseases. It is critical to further explore the impact of environmental exposure on health under the concept of exposure and enforce strict safety regulations and promote adequate workplace protection to reduce these risks. Training and regular health screenings can also contribute to early detection and prevention of occupational health issues.

## 4. Behavior risk factors

### 4.1. Role of diet in metabolic diseases and healthy aging

Diet plays a significant role in the onset and progression of metabolic diseases, with varying dietary habits yielding distinct impacts on these conditions. A well-balanced diet, rich in fruits, vegetables, whole grains, lean meats, and healthy fats, contributes to weight maintenance, blood sugar regulation, lowered cholesterol levels, and reduced risk of chronic diseases (69–71). Specific dietary factors, such as the overconsumption of sugary beverages and processed foods, elevate the risk of obesity and T2D. Conversely, diets high in fiber, antioxidants and omega-3 fatty acids, along with adequate intake of micronutrients and phytochemicals, can protect against these diseases and age-related decline (72–74). Interestingly, studies in invertebrates and rodents have found that calorie or diet restriction can slow down age-related diseases and prolong life expectancy (75). Simultaneously, Longo et al. (76) demonstrated that dietary interventions, such as intermittent fasting and protein restriction, can attenuate aging and extend a healthy lifespan (73). Furthermore, the Mediterranean diet has been proven to reduce mortality from cardiovascular disease (77, 78).

Moreover, diet significantly influences gut microbiota homeostasis, impacting energy metabolism and fat storage. Imbalances in gut flora, or dysbiosis, can lead to increased energy extraction and fat storage, contributing to obesity and diabetes (79, 80). Specifically, an increase in *Firmicutes* and a decrease in *Bacteroidetes* are commonly observed in these conditions (81, 82). In addition, certain potentially harmful bacterial groups may become overrepresented. For instance, the family *Enterobacteriaceae*, which includes potentially harmful species such as *Escherichia coli*, is often found to increase in metabolic diseases (83). These bacteria can lead to inflammation and further disrupt the balance in the gut microbiota. Conversely, beneficial bacteria may decrease in metabolic diseases. Examples include *Akkermansia muciniphila*, known to help maintain the health of the gut lining and *Bifidobacterium*, a genus often used in probiotics known for its health-promoting effects (84). These bacteria produce short-chain fatty acids that help regulate metabolism and inflammation, and their reduction can exacerbate metabolic issues. Positive dietary interventions, such as increasing the intake of prebiotic fibers and probiotic-rich foods, can help maintain healthy gut microbiota and promote healthy aging (85, 86). Additionally, age-related dysbiosis is linked to inflammation, promoting metabolic disorders and frailty, accelerating aging (87, 88). Understanding the intricate relationship between diet, gut flora, and overall health is crucial for mitigating metabolic diseases and fostering healthier aging outcomes.

### 4.2. Influence of physical activity and sedentary behavior on metabolic health

Declining activity and unhealthful changes in body composition are linked to aging. Physical activity and sedentary behavior have significant impacts on metabolic health.

Exercise prevents many chronic diseases and mitigates certain undesirable physiological changes brought on by aging. Interestingly, the impact of physical training on the reward system is a topic of growing interest. Engaging in regular physical activity reduces the motivation to seek unhealthy rewards like high-fat, sugary foods, and drugs. Additionally, it changes attitudes toward overeating, making individuals more mindful of their food choices. This transformation promotes healthier lifestyle preferences (89, 90).

Regular exercise also promotes glucose metabolism by increasing cells’ sensitivity to insulin, reducing the risk of developing T2D characterized by IR (91–93). Engaging in aerobic exercise training, such as brisk walking, jogging, swimming, wheel running, or cycling, increases energy expenditure, promotes cardiovascular health, and aids in weight management (94–96). Studies in the natural aging mouse model (C57BL/J6) have shown that early lifelong aerobic exercise training is crucial in preventing age-related issues, including muscle loss, decreased motility, and testicular atrophy, along with overall organ pathology (97). Testicular atrophy, the shrinking of testicles, can impact reproductive health. The findings highlight that aerobic exercise, when initiated early in life, can effectively mitigate these age-related challenges. Moreover, early-onset, lifelong running can inhibit inflammation, prevent multiple types of cancer, and prolong the healthy lifespan of naturally aging mice (97). The wheel running exercise can improve insulin sensitivity and treat IR in aging rats by restoring the role of hepatic insulin sensitizing substance. This substance is also known as hepatalin, a liver-produced hormone that enhances insulin sensitivity, particularly in skeletal muscle, promoting glucose storage as glycogen and contributing to the post-meal glucose disposal effect of insulin (53, 98). Resistance training, including weightlifting, improves metabolic health by enhancing muscle strength and mass, leading to an increased resting metabolic rate and insulin sensitivity (99, 100).

The World Health Organization recommends that adults engage in at least 150 min of moderate-intensity aerobic exercise or 75 min of vigorous exercise per week, or a combination of both (101, 102). It is well known that the level and intensity of physical activity decline as humans age, and during long-term aging, exercise affects changes in body weight and body composition in a gender-dependent manner (103). In women, estrogen plays a crucial role in regulating various aspects of metabolism. Estrogens regulate fat development by inhibiting preadipocyte differentiation, reducing lipolysis, favoring subcutaneous fat storage, and influencing energy expenditure. They boost the basal metabolic rate and can help maintain a healthy weight (104, 105). However, during menopause, declining estrogen levels can lead to increased abdominal fat accumulation. Studies in humans and rodents have shown that reduced estrogen production leads to IR and inflammation, and exercise relieves glucose intolerance and IR (106, 107). Moreover, estrogen deficiency in postmenopausal women and ovariectomized rodents results in increased respiratory exchange rate levels, decreased lipid oxidation, and increased carbohydrate oxidation during rest and exercise, accelerated cell senescence, and impaired bone formation (108, 109). Similarly, changes in male muscle mass, physical activity level, body weight, and fat percentage are closely related to testosterone. A decrease in testosterone levels will lead to an increase in body fat and a decrease in physical activity level (110, 111).

Conversely, a sedentary lifestyle characterized by prolonged sitting or inactivity is associated with an elevated risk of metabolic diseases, including obesity, T2D, and cardiovascular problems (112–114). Sedentary behavior leads to weight gain, reduced muscle mass, and impaired glucose and lipid metabolism (115, 116). It is important to note that even individuals who engage in regular exercise may still be at risk if they spend prolonged periods sitting or being inactive throughout the day (35). To promote metabolic health, it is crucial to incorporate regular physical activity into daily routines. This can include structured exercise sessions, such as workouts or sports, as well as simple lifestyle modifications like choosing to take the stairs instead of the elevator, walking or cycling for transportation, incorporating movement breaks during extended periods of sitting (117, 118). Elderly people are encouraged to walk to shopping when supermarkets are less than a kilometer away, as it is often the preferred mode of transportation over driving a car for their shopping needs (119).

### 4.3. Smoking and its association with metabolic diseases and aging

Tobacco smoking significantly elevates the risk for metabolic diseases and negatively affects the aging process. Cigarette smoke contains harmful compounds such as nicotine, carbon monoxide, and carcinogens that damage tissues and organs throughout the body. Smoking is linked to metabolic diseases like T2D, IR, obesity, and CVD, primarily due to nicotine’s contribution to IR and impaired glucose metabolism (120–123). In addition, smoking induces inflammation, oxidative stress, and endothelial dysfunction, leading to blood vessel damage and an increased risk of CVD (121, 124, 125). It also accelerates aging, causing premature skin aging, impaired wound healing, and elevating the risk of age-related conditions like osteoporosis and cognitive decline (126–128). Quitting smoking is crucial for reducing metabolic disease risk and promoting healthy aging. Immediate benefits post-cessation includes improved lung function, cardiovascular health, and overall well-being (129, 130).

### 4.4. Alcohol consumption and its impact on metabolic health in aging populations

Alcohol consumption’s impact on metabolic health hinges on individual factors and the quantity consumed. While moderate consumption might confer some cardiovascular benefits, excessive and chronic alcohol intake poses severe risks to metabolic health, particularly in aging populations (131). Heavy alcohol use promotes weight gain and obesity due to its caloric density and effects on appetite regulation. It also disrupts glucose metabolism, escalating the risk of IR and T2D (131–133). Toxic byproducts from alcohol metabolism can harm the liver, resulting in fatty liver disease, alcoholic hepatitis, and cirrhosis. Such chronic abuse hampers liver function, negatively impacting nutrient and medication metabolism and clearance, exacerbating metabolic health concerns (134, 135). Furthermore, alcohol can impede the absorption and utilization of essential nutrients, including vitamins and minerals, that are critical for maintaining metabolic health (136). Thus, for maintaining metabolic health in aging populations, it is recommended to moderate alcohol intake (131). Nonetheless, personal health conditions, medications, and other variables may necessitate adjustments to these guidelines, warranting consultation with a healthcare professional.

### 4.5. Sleep patterns and their relationship with metabolic diseases and aging

Sleep quality and patterns profoundly affect metabolic health and the aging process. Chronic sleep deprivation and disturbances are linked to increased metabolic disease risk and accelerated aging-related complications (137–139). Insufficient sleep and poor sleep quality can lead to obesity, T2D, CVD, and other metabolic disorders, primarily through disturbances in hormonal regulation of appetite and glucose metabolism (140–142). Sleep disruptions can also disrupt the body’s circadian rhythm, which regulates metabolic processes. These disturbances, such as shift work or irregular sleep schedules, contribute to metabolic dysregulation, hormonal imbalances, and increased inflammation (143, 144). Moreover, inadequate sleep impairs cognitive function and memory consolidation, which are essential for healthy aging (145, 146). Thus, promoting good sleep hygiene, including maintaining a consistent sleep schedule, creating a comfortable sleep environment, and using relaxation techniques, is crucial. Ensuring sufficient sleep duration, typically 7–9 h for adults, and seeking professional help for sleep disorders can support metabolic health and healthy aging.

### 4.6. The role of psychological stress and mental health in metabolic diseases and aging

Psychological stress and mental health significantly influence metabolic diseases and aging. Stress, depression, anxiety, and other mental health conditions are linked with metabolic diseases and can hasten the aging process (147–150). Stress and negative emotions can incite unhealthy behaviors such as overeating and inactivity, leading to obesity and related metabolic disorders (151, 152). Moreover, chronic stress disrupts hormonal balance, impairing the body’s ability to regulate blood sugar and manage inflammation (153). Recognizing the interplay between mental and physical health, public health initiatives should equally emphasize mental well-being. Promoting stress management, access to mental health services, and lifestyle changes that foster overall health could enhance interventions targeting healthy aging and metabolic disease prevention.

## 5. Promoting public health for healthy aging

Healthy aging is the focus of the World Health Organization’s aging policy by 2030 (154). The development of clear and targeted public health interventions is essential for mitigating the rising prevalence of metabolic diseases and promoting healthy aging.

These interventions should tackle determinants of metabolic diseases such as unhealthy dietary patterns, sedentary lifestyles, tobacco use, and environmental factors to effectively reduce their incidence and complications (7, 10, 35, 121, 124). Long-term healthy aging approaches require prevention throughout the life course. Specific public health strategies include: building strong social support systems, promoting health literacy promotion and lifestyle awareness and health education for older adult that can empower individuals to prevent metabolic diseases (155). Policies facilitating access to affordable, nutritious food, such as nutrition education, labeling requirements, and restrictions on marketing unhealthy foods have shown effectiveness in preventing metabolic diseases (156, 157).

Promoting physical activity or community volunteer work through community initiatives, workplace wellness programs, and accessible recreational facilities can improve metabolic health by maintaining healthy weight and enhancing insulin sensitivity. Infrastructure that prioritizes active transportation, green spaces, and safe recreational areas can further promote activity and reduce sedentary behavior (158, 159). Additionally, lifelong health promotion and disease prevention activities can help prevent or delay the onset and progression of metabolic diseases. The government should enhance and improve the healthcare system and primary healthcare services. Implementing regular routine screenings for middle-aged and elderly individuals can lead to the early detection and timely intervention of metabolic diseases, thereby minimizing their consequences. Furthermore, providing long-term, effective treatment, nursing care, and psychological support for patients with advanced diseases can enhance their sense of social well-being (160). Health regulations are also critical for promoting healthy aging and metabolic disease prevention. Therefore, a combination of multi-sectoral initiatives can create an environment that supports healthy behavior and promotes public health and healthy aging.

This article has certain limitations. It offers a comprehensive overview of metabolic diseases, but it fails to carry out in-depth analysis of the molecular processes associated with healthy aging and account for individual differences and subtle nuances, such as genetic factors and socio-economic circumstances. These elements play a significant role in the risk and progression of these diseases. Moreover, due to the complexity of the system and confounding variables, determining the causality between environmental/behavioral factors and metabolic diseases presents a challenge. For a complete understanding of metabolic diseases and healthy aging, further research and personalized assessments are critically essential.

## 6. Conclusion

In summary, this review article emphasizes the important contribution of environmental and behavioral risk factors to the emergence of metabolic diseases and healthy aging. Key risk factors, including diet, physical activity, smoking, alcohol consumption, environmental pollutants, and sleep patterns, have been identified and linked to various metabolic disorders and age-related complications. The findings underscore the importance of public health interventions in addressing metabolic diseases and promoting healthy aging. Further research is needed to understand risk factor interactions, mechanisms, and intervention effects. Addressing metabolic diseases is vital for healthy aging, reducing chronic conditions, and improving quality of life. A holistic approach combining behavior changes, supportive environments, and public health interventions can lead to a healthier future with lower metabolic disease rates and improved well-being in aging populations.

## Author contributions

KZ: Conceptualization, Data curation, Methodology, Writing – original draft. YuM: Conceptualization, Data curation, Methodology, Writing – original draft. YL: Conceptualization, Data curation, Investigation, Methodology, Writing – original draft. YS: Conceptualization, Data curation, Investigation, Writing – review & editing. GX: Data curation, Investigation, Writing – review & editing. YaM: Data curation, Investigation, Conceptualization, Writing – review & editing. XS: Conceptualization, Funding acquisition, Supervision, Writing – review & editing. CK: Conceptualization, Data curation, Investigation, Methodology, Supervision, Validation, Writing – review & editing.
